# Living Cationic Polymerization of Silyl-Protected β-Methyl Vinyl Ethers (Propenyl Ethers): Synthesis of Hydroxy-Functional Polymers with High *T*_g_ and LCST-Type Thermoresponse

**DOI:** 10.3390/molecules30224345

**Published:** 2025-11-10

**Authors:** Kohei Watanabe, Ryuya Yamada, Takeshi Namikoshi

**Affiliations:** Energy and Environmental Engineering, Faculty of Engineering, School of Earth, Kitami Institute of Technology, Kitami 090-8507, Japan

**Keywords:** poly(vinyl ether)s, hydroxy groups, living cationic polymerization, silyl protecting groups, thermal properties, thermoresponsive polymers

## Abstract

Hydroxy-functional poly(propenyl ether)s are promising thermoresponsive materials; here we establish a controlled synthesis via living cationic polymerization of silyl-protected monomers. Among the silyl protecting groups examined, only *tert*-butyldiphenylsilyl (TBDPS) enabled living cationic polymerization. The living cationic polymerization of *tert*-butyldiphenylsiloxybutyl propenyl ether (TBDPSBPE) afforded a high-molecular-weight polymer (poly(TBDPSBPE)) with a narrow molecular weight distribution (*M*_n_ = 12,900; *M*_w_/*M*_n_ = 1.22). Additionally, chain propagation continued in monomer addition experiments, and the molecular weight increased further with a narrow molecular weight distribution, confirming the success of living cationic polymerization. Poly(TBDPSBPE) was successfully desilylated to afford poly(HBPE) with a narrow molecular weight distribution. Poly(HBPE) exhibited a glass transition temperature (*T*_g_) of 44 °C, 82 °C higher than that of the corresponding polymer without β-methyl groups, poly(HBVE). The enhanced thermal properties of poly(HBPE) were attributed to the steric hindrance of the β-methyl group, which fixes the position of the hydroxy group and allows stronger hydrogen bonding. To investigate the aqueous thermoresponse, a hydroxylated analog with a shorter side-chain spacer (poly(HPPE)) was synthesized, and poly(HPPE) exhibited lower critical solution temperature (LCST)-type phase separation in water with a cloud-point temperature (T_cp_) of 6 °C, showing reversible transitions with thermal hysteresis.

## 1. Introduction

Vinyl ethers are representative monomers of cationic living polymerization [1,2,3]. In addition to simple alkyl vinyl ethers, vinyl ethers with a variety of functional groups, including oxygen-containing groups such as ether [4] and ester [5], and nitrogen-containing groups such as phthalimide [6] and urethane [7], can also undergo polymerization. For vinyl ethers with sulfur-containing functional groups, the cationic polymerization of alkyl sulfides results in a termination rection because the sulfur atom attacks the generated carbocation; nevertheless, living-like polymerization has been successfully achieved with phenyl sulfides [8]. When a vinyl ether with a hydroxy group undergoes cationic polymerization, the carbocation is nucleophilically attacked by the electron-rich hydroxy group to form a polymer with acetal bonds or a cyclic oligomer; therefore, poly(vinyl ether)s with hydroxy groups in the side chains cannot be directly synthesized [9]. Instead, such polymers can be synthesized by polymerizing a vinyl ether in which the hydroxy groups are protected with a protecting group, and then deprotecting the obtained polymer. Sugihara et al. reported the living cationic polymerization of a vinyl ether with *tert*-butyldimethylsiloxy (BMS) protecting groups and the subsequent deprotection of the obtained polymer to produce a poly(vinyl ether) with hydroxy groups in the side chains and a narrow molecular weight distribution [10].

Overall, cationic polymerization favors protect–deprotect strategies, whereas recent literature has established radical alternatives for direct vinyl-ether polymerization. Pioneering work by Sugihara et al. first demonstrated the direct radical (RAFT) polymerization of hydroxy-functional vinyl ethers (e.g., hydroxyethyl and hydroxybutyl vinyl ether), affording well-defined polymers and copolymers that exhibit lower critical solution temperature (LCST)-type phase separation in water [11,12]. Building on this foundation, the same group subsequently realized the direct radical homopolymerization of electron-rich vinyl ethers in aqueous LiOH via cooperative hydrogen bonding and Li^+^–π activation, and further extended the method to controlled RAFT, thereby demonstrating that even vinyl ethers are radically polymerizable under appropriately engineered conditions [13].

Although typical poly(alkyl vinyl ether)s are highly sticky liquid polymers with a low glass transition temperature (*T*_g_) [14], poly(alkyl vinyl ether)s with bulky side chains, such as tricyclodecane, have a higher *T*_g_ [15]. The *T*_g_ of vinyl polymers with β-methyl groups is higher than that of polymers without β-methyl groups; this trend also applies to poly(vinyl ether)s [16]. Although the cationic polymerization of propenyl ether, a vinyl ether with a β-methyl group, has long been reported [17], the living cationic polymerization of vinyl ethers is much more common. Nonetheless, various vinyl ethers with β-methyl groups, including *n*-butyl propenyl ether (NBPE) [18], isobutyl propenyl ether (IBPE) [19], and ethyl propenyl ether (EPE) [20,21], have been used as monomers for living cationic polymerization. Among these, NBPE and EPE with linear alkyl groups show higher reactivity than the corresponding vinyl ethers, indicating that the steric hindrance of the β-methyl groups does not affect the cationic polymerizability [21]. However, propenyl ethers with bulky substituents, such as *tert*-butyl propenyl ether (TBPE), are less reactive than the corresponding vinyl ethers [17]. While radical methods can polymerize several vinyl ethers, previous reports do not extend to β-methyl vinyl ethers (i.e., propenyl ethers). Accordingly, we investigated the living cationic polymerization of protected β-methyl monomers.

Recently, the living cationic polymerization of β-methyl-1-adamantyl vinyl ether (1-AdPE), in which a bulky adamantane group is directly bonded to an ether oxygen, was investigated with the aim of synthesizing a polyvinyl ether with a high *T*_g_. However, the resulting polymer was almost insoluble in organic solvents owing to its bulkiness and was hence difficult to characterize (see Appendix A). Although spacers between vinyl groups and bulky substituents are considered necessary to synthesize highly soluble poly(propenyl ether)s, the introduction of spacers leads to a decrease in *T*_g_ [15]. Therefore, to synthesize a poly(propenyl ether) with a higher *T*_g_, the polymerization of 4-hydroxybutyl propenyl ether (HBPE), a β-methyl vinyl ether (i.e., propenyl ether) bearing a flexible butyl spacer and a pendant hydroxy group, is suggested. The decreased bulkiness of these substituents is expected to facilitate polymerization, and hydrogen bonding is expected to increase the *T*_g_ of the resulting polymer. However, the cationic polymerization of vinyl ethers with hydroxy groups requires the use of a suitable protecting group.

**Figure 1:**
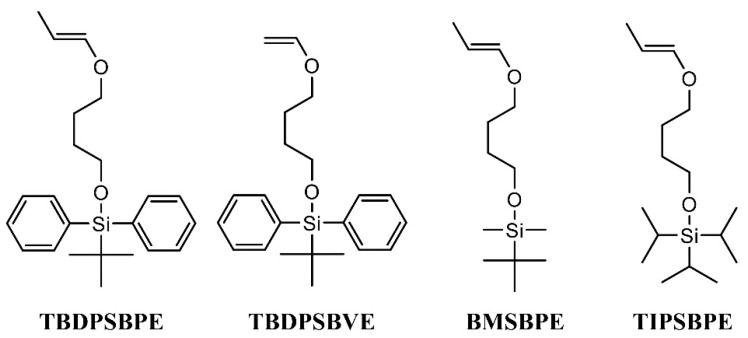
Structures of silyl-protected propenyl ethers bearing pendant hydroxy groups (β-methyl vinyl ethers) and corresponding vinyl ethers.

In this study, we synthesized HBPE and *tert*-butyldiphenylsilyloxybutyl propenyl ether (TBDPSBPE), in which the hydroxy group of HBPE is protected by a bulky silyl group (Figure 1). We also prepared the corresponding vinyl ether, i.e., *tert*-butyldiphenylsilyloxybutyl vinyl ether (TBDPSBVE), and compared their living cationic polymerization behavior. We then examined the polymerization properties of *tert*-butyldimethylsiloxybutyl propenyl ether (BMSBPE) and triisopropylsiloxybutyl propenyl ether (TIPSBPE), which have different substituents on the silyl group. Furthermore, to investigate the effect of bulky protecting groups and hydroxy groups after deprotection on the *T*_g_ of poly(β-methyl vinyl ether)s, we investigated the thermal properties of poly(TBDPSBPE) and poly(HBPE) and compared them with those of the corresponding poly(vinyl ether)s, poly(TBDPSBVE) and poly(4-hydroxybutyl vinyl ether) (poly(HBVE)). In addition to bulk thermal transitions, we also examined aqueous thermoresponsive LCST-type behavior of the deprotected polymers (poly(HBPE) and poly(propyl propenyl ether) (poly(HPPE))) bearing pendant –OH groups and compared them with poly(HBVE), to clarify how β-methyl substitution and side-chain design modulate both *T*_g_ and solution-phase transitions.

## 2. Results and Discussion

### 2.1. Polymerization of Propenyl Ethers with Silyl-Protected Hydroxy Groups

**Scheme 1:**
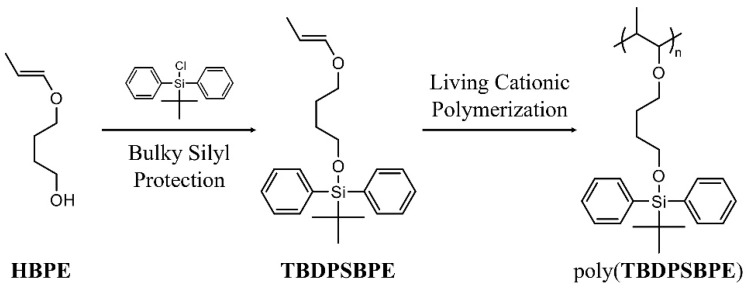
Synthesis of precursor monomers and subsequent living cationic polymerization.

HBPE, a β-methyl vinyl ether (propenyl ether) with a flexible alkyl chain and hydroxy group, was synthesized according to Scheme 1. Bulky *tert*-butyldiphenylsilane was then used as a protecting group for the hydroxy groups to form TBDPSBPE, and the living cationic polymerization of this monomer was investigated.

**Figure 2:**
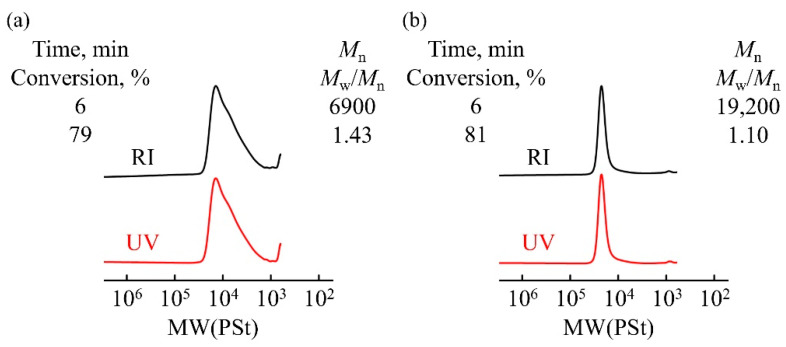
GPC traces of (**a**) poly(TBDPSBPE) and (**b**) poly(TBDPSBVE) prepared using HCl/ZnCl_2_ in toluene at −30 °C ([monomer]_0_ = 0.6 M, [HCl]_0_ = 0.5 mM, [ZnCl_2_]_0_ = 2.0 mM).

The polymerization of TBDPSBPE, a propenyl ether with a flexible butyl chain and a bulky protecting group, was carried out using the hydrogen chloride (HCl)/zinc chloride (ZnCl_2_) initiator system in toluene at −30 °C for 6 h ([TBDPSBPE]_0_ = 0.6 M, [HCl]_0_ = 0.5 mM, [ZnCl_2_]_0_ = 2.0 mM). As shown in Figure 2a, the gel permeation chromatography (GPC) trace of the polymer obtained by the polymerization of TBDPSBPE exhibits a broad peak in the low-molecular-weight region (*M*_n_ = 6900, *M*_w_/*M*_n_ = 1.43). Nevertheless, the chromatograms obtained using the RI and UV detectors appear similar, suggesting that the silyl protecting groups are not removed.

In contrast, the polymerization of the corresponding vinyl ether, TBDPSBVE, under the same conditions led to a polymer with a very narrow molecular weight distribution (*M*_w_/*M*_n_ = 1.10) without tailing (Figure 2b). These observations are consistent with the idea that steric hindrance from the β-methyl substituent in TBDPSBPE may suppress efficient propagation and promote processes such as chain transfer, leading to tailing and broader dispersity under these preliminary conditions. These data were collected as an initial screening of polymerizability and molecular-weight distribution; no kinetic analysis (*M*_n_ versus conversion) was performed under these conditions.

Based on prior reports on β-methyl vinyl ether polymerizations [21] and SnCl_4_-activated living polymerizations of vinyl ethers [22], we employed a 1-(isobutoxy)ethyl acetate (IBEA)/ ethylaluminum sesquichloride (Et_1.5_AlCl_1.5_)/tin tetrachloride (SnCl_4_) initiator system; in this design, Et_1.5_AlCl_1.5_ promotes the in situ acetate-to-chloride exchange of IBEA [22], and SnCl_4_ serves as the Lewis-acid activator.

Polymerization was carried out at −30 °C in toluene in the presence of ethyl acetate (AcOEt) ([TBDPSBPE]_0_ = 0.6 M, [IBEA]_0_ = 4.0 mM, [Et_1.5_AlCl_1.5_]_0_ = 4.0 mM, [SnCl_4_]_0_ = 5.0 mM, and [AcOEt]_0_ = 1.0 M).

**Figure 3:**
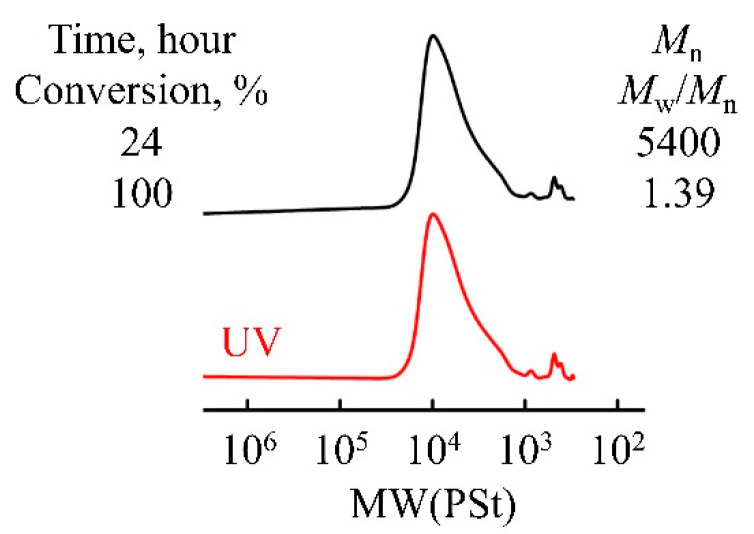
GPC traces of poly(TBDPSBPE) prepared using IBEA/Et_1.5_AlCl_1.5_/SnCl_4_ in toluene at −30 °C in the presence of AcOEt ([TBDPSBPE]_0_ = 0.6 M, [IBEA]_0_ = 4.0 mM, [Et_1.5_AlCl_1.5_]_0_ = 4.0 mM, [SnCl_4_]_0_ = 5.0 mM, [AcOEt]_0_ = 1.0 M).

As shown in Figure 3, the resulting polymer exhibits a low molecular weight, tailing, and a broad molecular weight distribution (*M*_w_/*M*_n_ = 1.39), likely because the steric hindrance of the β-methyl group inhibits the propagation reaction and causes a chain transfer reaction to occur.

**Figure 4:**
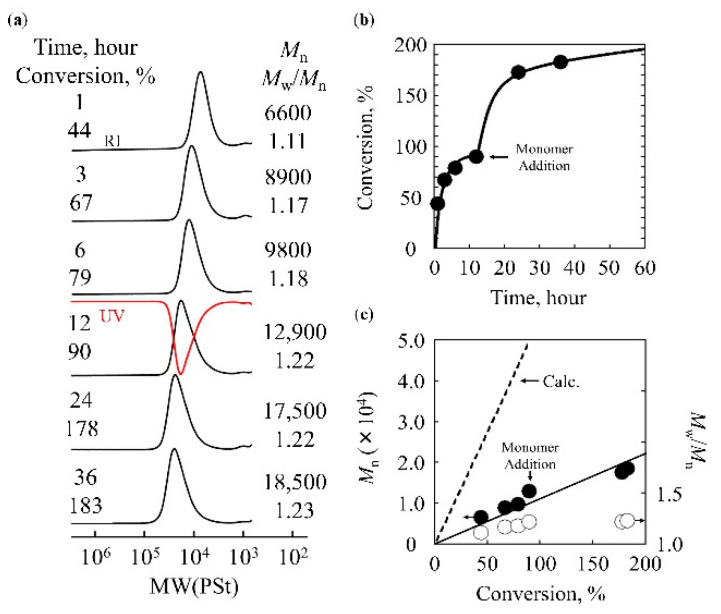
Living cationic polymerization of TBDPSBPE using IBEA/Et_1.5_AlCl_1.5_/SnCl_4_/AcOEt in toluene at −80 °C ([TBDPSBPE]_0_ = 0.6 M, [IBEA]_0_ = 4.0 mM, [Et_1.5_AlCl_1.5_]_0_ = 4.0 mM, [SnCl_4_]_0_ = 50 mM, [AcOEt]_0_ = 1.0 M, [TBDPSBPE]_add_ = 0.6 M): (**a**) GPC traces of the polymerization products at various stages; (**b**) time–conversion profile; (**c**) evolution of *M*_n_ and *M*_w_/*M*_n_ as a function of monomer consumption. Open symbols indicate aliquots taken during the initial polymerization stage (prior to additional monomer feed), while filled symbols denote samples taken after introducing additional monomer for chain extension. The solid line (“Calc.”) represents the theoretical *M*_n_ for a living polymerization, calculated based on the monomer/initiator ratio consumed at each sampling point (*M*_n_,calc = *M*_0_ × [monomer consumed]/[initiator] + *M*_end-groups_, where *M*_0_ is the molar mass of the repeating unit and *M*_end-groups_ is the combined mass of the α- and ω-chain ends). Because additional monomer was introduced to the same living chains, cumulative conversion exceeds 100%, as shown on the x-axis in panel (**c**).

To suppress side reactions, the polymerization temperature was lowered to −80 °C. As lowering the temperature was expected to significantly reduce the polymerization rate, the concentration of the activator (SnCl_4_) was increased to 50 mM ([TBDPSBPE]_0_ = 0.6 M, [IBEA]_0_ = 4.0 mM, [Et_1.5_AlCl_1.5_]_0_ = 4.0 mM, [SnCl_4_]_0_ = 50 mM, [AcOEt]_0_ = 1.0 M). Consequently, the monomer conversion increased. Moreover, the molecular weight increased linearly with the monomer conversion, but the molecular weight distribution remained narrow (Figure 4). In Figure 4c, open symbols denote aliquots taken during the initial polymerization stage, whereas filled symbols denote samples taken after additional monomer was fed for the chain-extension experiment. The solid line (“Calc.”) is the theoretical *M*_n_ expected for a living system, calculated from the cumulative monomer-to-initiator ratio consumed at each sampling point. Because the second stage involves feeding more monomers to the same living chains, the cumulative monomer consumption can exceed the initial 100% charge, as plotted in Figure 4c. In the GPC measurements, the chromatograms obtained using the RI and UV detectors overlapped. Furthermore, when additional monomer was introduced at a later stage in the polymerization and polymerization was resumed, the number-average molecular weight increased further while the molecular weight distribution remained narrow, and the peak maximum shifted toward the high-molecular-weight region. In this chain-extension experiment, essentially all of the initially charged monomer was consumed, and the subsequently added monomer also underwent further propagation on the same chains. 100% conversion of the additionally added monomer was observed under the same conditions. These results confirm that the living cationic polymerization of TBDPSBPE was achieved using the IBEA/Et_1.5_AlCl_1.5_/SnCl_4_ initiator system in toluene at −80 °C in the presence of AcOEt. In contrast to the preliminary HCl/ZnCl_2_ experiments summarized in Figure 2, Figure 4 presents the optimized SnCl_4_-based conditions, under which we monitored *M*_n_ as a function of monomer conversion and demonstrated successful chain extension. Together, these data establish that TBDPSBPE undergoes living cationic polymerization under the optimized conditions.

### 2.2. Structure of Poly(TBDPSBPE)

**Figure 5:**
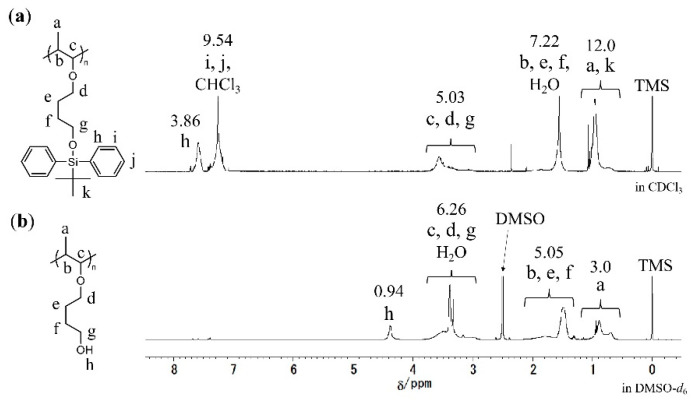
^1^H NMR spectra of (**a**) poly(TBDPSBPE) in CDCl_3_ and (**b**) the corresponding polymer after desilylation in DMSO-*d*_6_.

**Figure 6:**
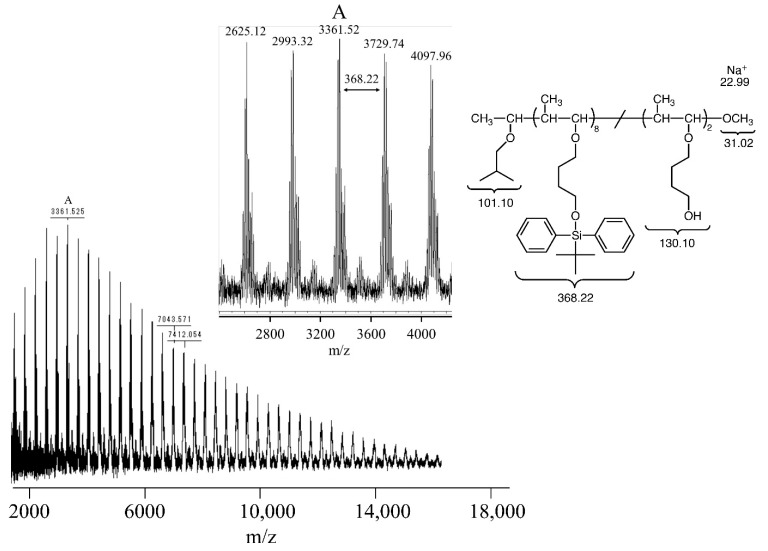
MALDI-TOF MS spectra of poly(TBDPSBPE) prepared using IBEA/Et_1.5_AlCl_1.5_/SnCl_4_ at −80 °C in the presence of AcOEt ([TBDPSBPE]_0_ = 0.08 M, [IBEA]_0_ = 4.0 mM, [Et_1.5_AlCl_1.5_]_0_ = 4.0 mM, [SnCl_4_]_0_ = 50 mM, [AcOEt]_0_ = 1.0 M).

In Figure 5a, the ^1^H NMR spectrum of the obtained polymer is consistent with the proposed structure of poly(TBDPSBPE), suggesting minimal deprotection during living cationic polymerization. For MALDI-TOF MS measurements, low-molecular-weight poly(TBDPSBPE) was synthesized. The MS spectrum displayed peaks separated by 368.22 mass units (Figure 6), corresponding to a single repeat unit. An end-group analysis indicated the presence of an initiator-derived isobutyl vinyl ether at the α terminus and a methanol-derived methoxy group at the ω terminus. A major ion at *m*/*z* 3361.52 (peak A) was assignable to a 10-mer that had lost two TBDPS groups (calcd *m*/*z* 3361.06; Δ = +0.46 Da), implying that at least two silyl groups were cleaved within that chain. Importantly, higher-*m*/*z* members of the same series, including ions that were assignable to the 20-mer and 21-mer, exhibited identical end groups and the loss of exactly two TBDPS groups, with increments of 368.22 Da, consistent with chain growth proceeding without additional deprotection at higher degrees of polymerization. Collectively, these observations suggest that silyl-group elimination, if it occurs, likely takes place at an early stage of polymerization, while subsequent propagation proceeds without further loss. A complementary GPC analysis of the bulk sample (Figure 4) showed a unimodal and narrow chromatogram with no low-*M*_n_ shoulder, supporting a high degree of livingness and suggesting that frequent silyl-group elimination does not occur during polymerization.

### 2.3. Effect of Silyl Group Substituents

**Figure 7:**
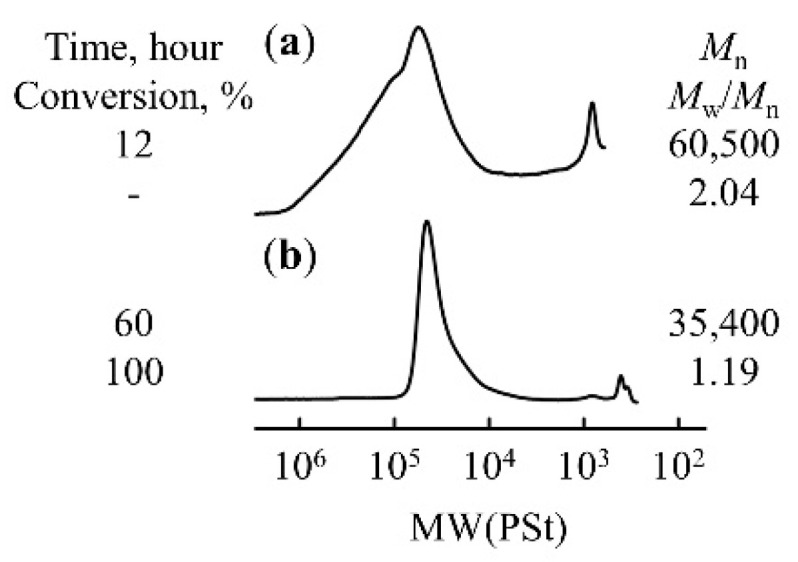
GPC traces of (**a**) poly(BMSBPE) and (**b**) poly(TIPSBPE) prepared using IBEA/Et_1.5_AlCl_1.5_/SnCl_4_ in toluene at −80 °C in the presence of AcOEt ([monomer]_0_ = 0.6 M, [IBEA]_0_ = 4.0 mM, [Et_1.5_AlCl_1.5_]_0_ = 4.0 mM, [SnCl_4_]_0_ = 5.0 mM, [AcOEt]_0_ = 1.0 M).

The effect of varying the silyl group substituent on the polymerization of propenyl ethers bearing silyl-protected hydroxy groups was investigated. Using the optimal conditions for the polymerization of TBDPSBPE (the IBEA/Et_1.5_AlCl_1.5_/SnCl_4_ initiator system at −80 °C in the presence of AcOEt), propenyl ethers with hydroxy groups protected by BMS and the triisopropylsilyl (TIPS) group were polymerized. The polymerization of BMSBPE produced a polymer with a high molecular weight (*M*_n_ = 60,500), but the molecular weight distribution was broad (*M*_w_/*M*_n_ = 2.04), suggesting that deprotection and cross-linking reactions between polymers occurred during polymerization. The polymerization of TIPSBPE with more bulky silyl groups resulted in an increase in the molecular weight with increasing monomer conversion, yielded high-molecular-weight materials with a narrow molecular weight distribution (Figure 7). However, in monomer addition experiments, the chromatograms became bimodal, indicating a lower degree of livingness than in the case of TBDPSBPE (Figure S8, Supporting Information).

### 2.4. Desilylation

**Scheme 2:**
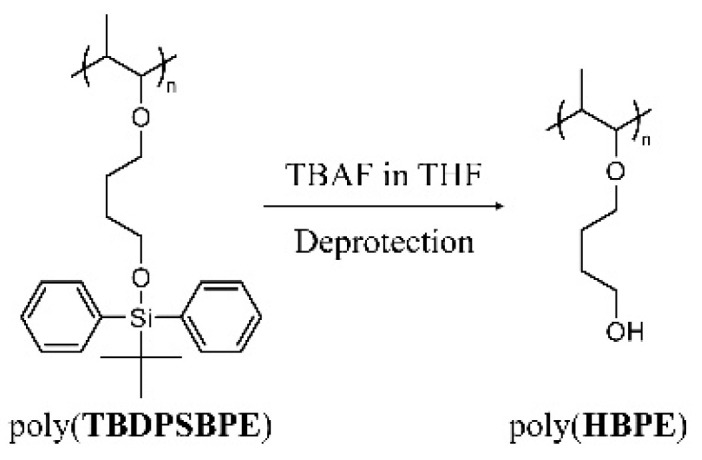
Synthesis of poly(HBPE) by the desilylation of poly(TBDPSBPE).

The desilylation of poly(TBDPSBPE) was performed as shown in Scheme 2. As shown by the ^1^H NMR spectra before and after desilylation (Figure 5), the phenyl group peak of the silyl group in the polymer side chain, which was observed at 7–8 ppm before desilylation, almost disappears after desilylation. A comparison of the peak intensities of the butyl groups in the side chains (e, f) and the methine group in the main chain (b) relative to the phenyl group of the silyl group (g) confirmed 99% desilylation. The peak intensity ratios in the ^1^H NMR spectrum were consistent with the structure of poly(HBPE), indicating the successful formation of the desired desilylated polymer.

Following NMR verification, the samples were analyzed by GPC. Because the desilylated polymer was no longer soluble in THF, measurements were carried out in DMF, and the results are summarized in Table 1. After desilylation, the apparent *M*_n_ of the hydroxylated poly(propenyl ether) dropped from 19,600 to 7500, i.e., by ~61.7%. This decrease is quantitatively reasonable: the repeat-unit mass in this series decreases from 368.592 Da (TBDPS-protected) to 130.187 Da (deprotected), corresponding to an expected mass loss of ~64.7% if the degree of polymerization is retained. In contrast, for the analogous vinyl ether series, *M*_n_ drops from 19,200 to 3500 (~81.8%) upon deprotection, which is substantially larger than the ~67.2% decrease predicted from the change in repeat-unit mass (354.565 → 116.160 Da). We attribute this additional apparent decrease to the GPC measurement conditions: after desilylation, the hydroxyl-rich vinyl ether polymer must be analyzed in DMF, where strong interactions with the GPC column delay elution relative to polystyrene standards of comparable true chain length. When conventional polystyrene calibration is applied under these DMF GPC conditions, this later elution leads to an underestimation of *M*_n_. A similar underestimation was observed for poly(HBVE) under the same DMF GPC conditions. Importantly, the dispersity of the desilylated samples remains narrow, indicating that the chain-length distribution is preserved. Finally, because the desilylated hydroxylated polymers are not soluble in THF, we also recorded GPC traces in DMF for poly(HBVE), poly(HBPE), and poly(HPPE); these data are provided in the Supporting Information (Figure S13). In DMF, each desilylated polymer displayed a single, unimodal elution peak without evidence of large low-molecular-weight shoulders. This behavior supports our interpretation that (i) the desilylation step does not generate extensive backbone scission or multiple polymer populations, and (ii) the very low apparent *M*_n_ values reported for the hydroxylated polymers arise mainly from DMF/column interactions and polystyrene calibration rather than from broad degradation.

### 2.5. Thermal Properties

#### 2.5.1. Glass Transition (by Differential Scanning Calorimetry (DSC))

The thermal properties of the synthesized polymers (*T*_g_, glass transition temperature, and *T*_d_, thermal decomposition temperature) were measured, as summarized in Table 1. *T*_g_ is the glass transition temperature, defined as the midpoint of the step change in heat flow observed during the second heating cycle in DSC measurements under N_2_. *T*_d_ is the temperature of decomposition, defined as the temperature at which a 5 wt% mass loss is observed in TGA measurements under N_2_. The thermal properties were compared with those of the corresponding poly(vinyl ether)s with similar structures to investigate the effect of the β-methyl group on the hydroxy and bulky silyl groups in the side chain.

The *T*_g_ values of poly(TBDPSBPE) and poly(HBPE), which are β-methyl-substituted poly(vinyl ether)s, were 6.5 and 77 °C, respectively, higher than those of poly(TBDPSBVE) and poly(HBVE), which are the corresponding polymers without β-methyl groups. These results confirm that the thermal properties were enhanced by the presence of the β-methyl groups. *T*_g_ also changed significantly before and after desilylation. The *T*_g_ of poly(HBVE), which bears pendant hydroxy groups, was 33 °C lower than that of its silyl-protected precursor poly(TBDPSBVE), indicating a large *T*_g_ decrease upon desilylation in the β-unsubstituted series. In contrast, the *T*_g_ of poly(HBPE), which bears pendant hydroxy groups, was 37.5 °C higher than that of its silyl-protected precursor poly(TBDPSBPE), indicating a large *T*_g_ increase upon desilylation in the β-methyl series. These different trends, despite the elimination of the same protecting group to form a hydroxy group, may be due to restriction of main-chain rotational motion by the β-methyl group, which would in turn strengthen intermolecular hydrogen bonding between hydroxy groups and thus increase *T*_g_. We also compared the *T*_d_ values within each series. In both the β-unsubstituted (vinyl ether) and β-methyl (propenyl ether) series, the silyl-protected precursors showed higher *T*_d_ values than their corresponding desilylated hydroxylated polymers, consistent with the greater thermal robustness of the bulky silyl substituent compared to free pendant hydroxy groups. At the same time, comparison within each protected/desilylated pair indicates that the β-methyl–substituted polymers generally maintain *T*_d_ values that are comparable to, or slightly higher than, those of the β-unsubstituted analogs, suggesting that introducing a β-methyl substituent can enhance *T*_g_ without causing a marked penalty in thermal stability [16]. Representative DSC curves for *T*_g_ and TGA curves for *T*_d_ are shown in the Supporting Information (Figures S14 and S15).

#### 2.5.2. Thermoresponsive Behavior in Water (LCST)

**Figure 8:**
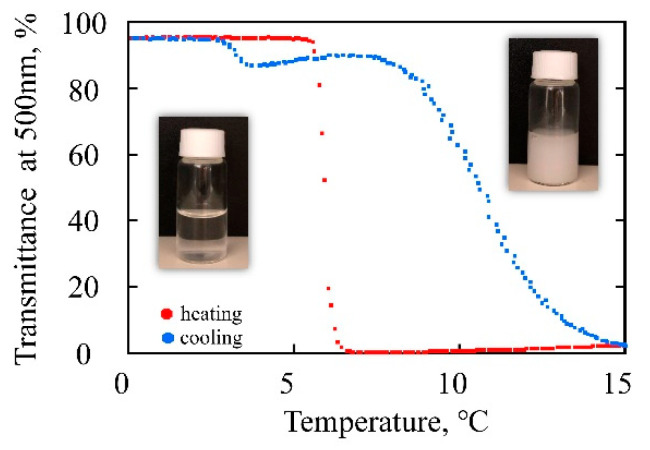
Temperature dependence of the transmittance at 500 nm of 0.25 wt% aqueous solutions of poly(HPPE): heating (red) and cooling (blue). The rate of transmittance measurement was 1.0 °C/min.

Hydroxyl-functionalized poly(hydroxybutyl vinyl ether) (poly(HBVE)) is known to exhibit an LCST in water [10]. Because our poly(HBPE) also bears pendant hydroxyl groups, we first evaluated its solubility in various solvents. After the desilylation of the precursor poly(TBDPSBPE), the resulting poly(HBPE) became significantly less soluble in common organic solvents such as THF and toluene and was only soluble in more polar media (e.g., DMF, DMSO), indicating a pronounced change in solubility profile. However, poly(HBPE) remained insoluble in water (Table 2), which we attributed to the increased hydrophobicity imparted by the β-methyl substituent on the backbone. To balance the hydroxyl group and methylene units similarly to poly(HBVE), we synthesized poly(propyl propenyl ether) (poly(HPPE)) by preparing poly(TBDPSPPE). In this case, the poly(TBDPSPPE) precursor was not fully soluble in common organic GPC eluents such as THF; only the dissolved portion could be analyzed by GPC, and an insoluble fraction remained. Because this limited solubility complicated full characterization of the protected polymer, we proceeded directly to the desilylation step, which was quantitative (>99%) as confirmed by ^1^H NMR (see Supporting Information: the stepwise synthesis of TBDPSPPE and ^1^H NMR spectra of the intermediates (Figures S6–S8), and GPC chromatograms and the ^1^H NMR spectrum of poly(HPPE) after desilylation (Figures S11 and S12)). Aqueous solutions of poly(HPPE) displayed LCST-type phase separation. At ~0 °C, the solution was clear but turned turbid at ambient temperature. Upon heating from 0 °C at 0.1 °C min^−1^, the transmittance at 500 nm sharply decreased at the cloud point temperature (T_cp_) of 6 °C (Figure 8, heating). Upon cooling, the transmittance recovered more gradually, and the clearing temperature (T′_cp_) appeared at a higher temperature than T_cp_, indicating pronounced thermal hysteresis (Figure 8, cooling). While typical LCST polymers show steep transitions with minimal hysteresis, poly(HPPE) exhibits slow rehydration. One possible explanation is that the β-methyl substituent imposes steric constraints that slow disaggregation and re-solubilization. This mechanism remains untested; we plan to assess it using positional isomers (e.g., α- vs. β-substitution) and related structural analogs. The substantially lower T_cp_ of poly(HPPE) (6 °C) compared to that of poly(HBVE) (~42 °C) further supports the observation that the β-methyl group enhances hydrophobicity, thereby decreasing the phase-separation temperature.

## 3. Materials and Methods

### 3.1. Materials

IBEA was synthesized according to the literature and subdivided into ampoules [23]. Toluene (Wako, Osaka, Japan; super dehydrated) and diethyl ether (Wako; super dehydrated) were purified by passage through solvent purification columns (Glass Contour, AS ONE, Osaka, Japan) before use as polymerization solvents. HCl (Sigma-Aldrich, St. Louis, MO, USA; 4.0 M solution in 1,4-dioxane), ZnCl_2_ (Sigma-Aldrich; 1.0 M solution in diethyl ether), Et_1.5_AlCl_1.5_ (Kanto Chemical, Tokyo, Japan; 1.82 M solution in toluene), SnCl_4_ (Sigma-Aldrich; 1.0 M solution in CH_2_Cl_2_), and AcOEt (Wako; super dehydrated) were used as polymerization reagents without further purification. 1.4-Butanediol (Wako, Osaka, Japan), allyl bromide (Sigma-Aldrich, St. Louis, MO, USA), sodium hydroxide (Wako, Osaka, Japan), tetra-*n*-butylammonium bromide (TBAB, Wako, Osaka, Japan), and *tert*-butyldiphenylchlorosilane (Sigma-Aldrich, St. Louis, MO, USA) were used for monomer synthesis, and all other reagents were also used without further purification.

### 3.2. Synthesis of 4-Hydroxybutyl Allyl Ether (HBAE)

HBAE was synthesized by the reaction of 1,4-butanediol with allyl bromide. 1,4-Butanediol (19.80 mL, 0.224 mol), allyl bromide (19.17 mL, 0.226 mol), TBAB (1.4 g, 4.34 mmol), sodium hydroxide (8.8 g, 0.22 mol), and toluene (60 mL) were added to a 300 mL three-necked round-bottom flask fitted with a reflux condenser, which was then purged with nitrogen. After heating at 70 °C for 10 h with stirring, the reaction mixture was washed with water. The aqueous phase was then extracted with toluene, and the combined organic layers were dried and concentrated to afford HBAE as a colorless transparent liquid (yield: 19.84 g, 68%). ^1^H NMR (CDCl_3_, ppm): 1.65–1.73 (m, 4H, –O–CH_2_CH_2_CH_2_CH_2_–OH), 2.42 (t, 1H, –O–CH_2_CH_2_CH_2_CH_2_–OH), 3.45, 3.48, 3.64 (m, 4H, –O–CH_2_CH_2_CH_2_CH_2_–OH), 3.97 (t, 2H, CH_2_=CHCH_2_–O–), 5.15–5.20, 5.25–5.30 (m, 2H, CH_2_=CHCH_2_–O–), 5.92 (m, 1H, CH_2_=CHCH_2_–O–) (shown in Figure S1).

### 3.3. Synthesis of HBPE

HBPE was synthesized by a ruthenium-catalyzed reaction of HBAE with methanol [24]. An autoclave reactor with a polytetrafluoroethylene-lined vessel was charged with HBAE (19.84 g, 0.152 mol), methanol (18.47 mL, 0.455 mol), sodium carbonate (0.806 g, 7.6 mmol), and RuCl_2_(PPh_3_)_3_ (1.46 g, 1.52 mmol) and then purged with nitrogen. The mixture was heated at 120 °C for 3 h with stirring. After filtering the reaction mixture to remove sodium carbonate and RuCl_2_(PPh_3_)_3_, the product was isolated by column chromatography using a mixture of AcOEt and hexane (1/2, *v*/*v*) and then distilled over calcium hydride under reduced pressure to obtain HBPE as a colorless liquid (yield: 15.85 g, 80%; density: 0.9179 g/mL; bp: 48 °C/3 mmHg). The product contained a mixture of Z/E isomers, with a ratio of approximately 65:35 as determined by integration of the vinyl proton signals in the ^1^H NMR spectrum (Figure S2a). No attempt was made to separate the isomers prior to the subsequent reactions. ^1^H NMR (CDCl_3_, ppm): 1.50, 1.53 (d, 3H, CH_3_CH=CH–O–), 1.58–1.69 (m, 4H, –O–CH_2_CH_2_CH_2_CH_2_–OH), 2.51 (s, 1H, –O–CH_2_CH_2_CH_2_CH_2_–OH), 3.61, 3.72 (m, t, 4H, –O–CH_2_CH_2_CH_2_CH_2_–OH), 4.35, 4.74 (m, 1H, CH_3_CH=CH–O–), 5.90, 6.16 (dd, 1H, CH_3_CH=CH–O-) (shown in Figure S2a); ^13^C NMR (CDCl_3_, ppm): 9.22, 12.58 (CH_3_CH=CH–O–), 25.90, 26.35, 29.41 (–O–CH_2_CH_2_CH_2_CH_2_–OH), 62.40 (–O–CH_2_CH_2_CH_2_CH_2_–OH), 68.99, 71.87 (–O–CH_2_CH_2_CH_2_CH_2_–OH), 98.85, 101.19 (CH_3_CH=CH–O–), 145.37, 146.36 (CH_3_CH=CH–O–) (shown in Figure S2b). Notably, after isomerization and purification, the allyl-ether signals (CH_2_=CH–CH_2_–O–; ^1^H NMR) were no longer observed (Figure S2a), indicating the complete consumption of the allyl precursor. The propenyl ether geometry (E/Z) remained unchanged during silylation, as confirmed by the preserved chemical shifts in ^1^H NMR (Figure S3a). In contrast, these signals were no longer detectable after polymerization, suggesting successful incorporation of the propenyl moieties into the polymer backbone.

### 3.4. Synthesis of TBDPSBPE

TBDPSBPE was synthesized by reacting HBPE with *tert*-butyldiphenylchlorosilane [25]. A solution of *tert*-butyldiphenylchlorosilane (15.0 mL, 0.0584 mol) in *N*,*N*-dimethylformamide (DMF; 17.5 mL) was added dropwise to a mixture of HBPE (9.22 g, 0.0708 mol), imidazole (8.75 g, 0.129 mol), and DMF (17.5 mL) at 0 °C under nitrogen. The mixture was stirred at ambient temperature (rt; uncontrolled, ca. 22–26 °C) for 6 h and then washed with water. The crude monomer was distilled over calcium hydride under reduced pressure to afford TBDPSBPE as a colorless liquid (yield: 18.53 g, 86%; density: 0.9850 g/mL; bp: 160 °C/2 mmHg). The purified monomer retained the original Z/E isomer ratio (~63:37) of HBPE, as evidenced by consistent ^1^H NMR signals (Figure S3a). ^1^H NMR (CDCl_3_, ppm): 1.08 (s, 9H, –Si–C(CH_3_)_3_), 1.58 (d, 3H, CH_3_CH=CH–O–), 1.67 (m, 2H, –O–CH_2_CH_2_CH_2_CH_2_–O–Si–), 1.74 (m, 2H, –O–CH_2_CH_2_CH_2_CH_2_–O–Si–), 3.64, 3.73 (t, m, 4H, –O–CH_2_CH_2_CH_2_CH_2_–O–Si–), 4.39, 4.77 (m, 1H, CH_3_CH=CH–O–), 5.95, 6.22 (dd, 1H, CH_3_CH=CH–O–), 7.39–7.45, 7.70 (m, 10H, –Si–(C_6_H_5_)_2_) (shown in Figure S3a); ^13^C NMR (CDCl_3_, δ): 9.31, 12.71 (CH_3_CH=CH–O–), 19.31–29.12 (–O–CH_2_CH_2_CH_2_CH_2_–O–Si–), 19.30, 26.96 (–Si–C(CH_3_)_3_), 63.64 (–O–CH_2_CH_2_CH_2_CH_2_–O–Si-), 69.02, 71.90 (–O–CH_2_CH_2_CH_2_CH_2_–O–Si–), 98.46, 100.92 (CH_3_CH=CH–O–), 127.50–135.66 (–Si–(C_6_H_5_)_2_), 145.37, 146.36 (CH_3_CH=CH–O–) (shown in Figure S3b); HRMS (EI): *m*/*z* calculated for C_19_H_23_O_2_Si [M-C(CH_3_)_3_]^+^: 311.14673; observed: 311.14681.

After the allyl-to-propenyl isomerization step, the crude mixture was subjected to column chromatography followed by reduced-pressure distillation to isolate the pure propenyl ether monomer (HBPE) and its silyl-protected analog (TBDPSBPE), which were then used for polymerization. After isomerization and purification, the characteristic allylic CH_2_=CH–CH_2_–O– ^1^H resonances of the starting allyl ether precursor were no longer observed in the ^1^H NMR spectrum, indicating consumption of the allyl species prior to polymerization. The purified, silyl-protected β-methyl propenyl ether monomer was further analyzed by EI-MS (direct injection). Under EI ionization, the *tert*-butyldiphenylsilyl substituent undergoes characteristic fragmentation, including loss of the *tert*-butyl group, so the most intense ions correspond to diagnostic fragment ions rather than an intact molecular ion. The resulting fragment pattern is consistent with the assigned silyl-protected β-methyl propenyl ether structure. Although EI-MS alone cannot fully exclude the presence of very minor allyl-derived species, we did not detect any additional, clearly distinguishable series of fragment ions that would suggest a substantial allyl-ether–type component.

These spectral data are provided in the Supporting Information and discussed below.

### 3.5. Polymerization Procedure

The polymers were synthesized using the following procedure. Polymerization was carried out under nitrogen in a reactor composed of a glass tube equipped with a three-way stopcock, as reported previously [26]. The initiator (0.5 mL) and activator (0.5 mL) were sequentially added to a 4.0 mL toluene solution of the monomer containing AcOEt as an added base (except when HCl/ZnCl_2_ was used as the initiator system), cooled to the polymerization temperature. Polymerization was terminated by adding methanol (2.0 mL) containing a small amount of triethylamine. Subsequently, the polymerization solution was diluted with toluene and washed with water; then, the solvent was removed using a rotary evaporator, and the product was dried *in vacuo*.

### 3.6. Synthesis of Poly(HBPE) by Desilylation [27]

Poly(TBDPSBPE) was purified by dissolution in tetrahydrofuran (THF) and precipitation in methanol, and the precipitate was isolated by filtration The purified polymer (1.12 g, *M*_n_(calc.) = 55,286) and tetrabutylammonium fluoride (TBAF; 1.0 M in THF solution, 3.6 mL) were placed in a flask and allowed to react for 6 h at room temperature. After removing THF, the polymer was purified by dialysis (MWCO 3500, Funakoshi Co., Ltd., Tokyo, Japan) in methanol for 1 week. The other polymers with silyl-protected hydroxy groups were deprotected in the same manner.

### 3.7. Polymer Characterization

Monomer conversion was determined by ^1^H NMR spectroscopy from the ratio of the peak of the propenyl group (CH_3_–CH=CH–O–) of the residual monomer to the peak of the silyl protecting group in the side chains. The molecular weight (*M*_w_) and molecular weight distribution (*M*_w_/*M*_n_) of the obtained polymers were determined by GPC using a calibration curve prepared with polystyrene standards *(M*_w_: 500, 1050, 2630, 5970, 9100, 18,100, 96,400, and 355,000). The GPC system consisted of a L-7100 pump (HITACHI, Tokyo, Japan), one KF-802 column, three LF-804 columns (Shodex, Tokyo, Japan), an refractive index detector (RI detector) RI-8020 (TOSOH, Tokyo, Japan), and a UV detector SPD-10A (Shimadzu, Kyoto, Japan). GPC measurements were carried out using THF at a flow rate of 1.0 mL/min and 40.0 °C. GPC analysis of polymers bearing hydroxy groups was performed using a CCPS pump (TOSOH, Tokyo, Japan) and an RI-8020 refractive index detector (TOSOH, Tokyo, Japan). The measurements were conducted in DMF as the eluent, at a flow rate of 0.6 mL/min and 40.0 °C, using an Asahipak GF-7M HQ column (Shodex, Tokyo, Japan). The ^1^H (600 MHz) NMR spectra were recorded on a JNM-ECA600 instrument (JEOL, Tokyo, Japan) at room temperature against tetramethylsilane as the internal standard. Matrix-assisted laser desorption/ionization time of flight mass spectrometry (MALDI-TOF MS) was performed using a Ultraflex III instrument (Bruker Daltonics, Massachusetts, USA) with dithranol as the matrix and sodium trifluoroacetate as the cationizing agent. The *T*_g_ value of each polymer was determined by DSC using a DSC-60 instrument (Shimadzu, Kyoto, Japan) and an aluminum sample pan under a nitrogen atmosphere. The heating and cooling rates for the first cycle were 10 °C/min and those for the second cycle were 5 °C/min. The thermal decomposition temperature (*T*_d_) of each polymer was measured under a nitrogen atmosphere using a TG-8121 thermal analyzer (Rigaku, Tokyo, Japan) with a platinum sample pan and alumina as the reference sample. The temperature was increased from room temperature to 700 °C at a rate of 10 °C/min. Aqueous polymer solutions were prepared by dissolving the polymer in Milli-Q water (18.2 MΩ·cm) (Merck, Darmstadt, Germany) and adjusting to the desired concentration. Phase separation was evaluated by measuring the transmittance at 500 nm through a 1 cm glass cell during heating and cooling scans from 0 to 15 °C at 0.1 °C min^−1^. Transmittance was recorded on a V-770DS UV–Vis spectrophotometer (JASCO, Tokyo, Japan) equipped with a Peltier-type thermostatted cell holder (ETCR-762, JASCO, Tokyo, Japan). The LCST was defined as the cloud-point temperature (T_cp_), corresponding to the temperature at which the transmittance at 500 nm decreases to 50% on heating; the corresponding value measured during cooling is denoted as T′_cp_.

## 4. Conclusions

The living cationic polymerization of a propenyl ether with a flexible butyl chain and a bulky TBDPS group protecting the hydroxy group was achieved at −80 °C using an IBEA/Et_1.5_AlCl_1.5_/SnCl_4_ initiator system. A poly(propenyl ether) with hydroxy groups on the side chains was synthesized by the desilylation of the resulting polymer with TBAF. *T*_g_ was markedly increased by introducing a β-methyl group into the poly(vinyl ether) main chain, while *T*_d_ was maintained at comparable or higher levels. The significant increase in *T*_g_ was attributed to the suppression of the rotational motion of the main chain and the strengthening of hydrogen bond interactions between the hydroxy groups. In water, poly(HBPE) was insoluble, whereas the shorter-spacer analog poly(HPPE) exhibited LCST-type phase separation (T_cp_ ≈ 6 °C) with reversible hysteresis (T′_cp_ > T_cp_). The molecular origin of this hysteresis remains unresolved and will be examined in future work using α-methyl analogs and related structural analogs.

## Figures and Tables

**Table 1:** Thermal properties of poly(propenyl ether)s and the corresponding poly(vinyl ether)s bearing bulky silyl protecting groups or pendant hydroxy groups.

	Poly(TBDPSBPE)	Poly(HBPE)	Poly(TBDPSBVE)	Poly(HBVE)
*M*_n_	19,600	7500 (−61.7%)	19,200	3500 (−81.8%)
*T*_g_ (°C)	1.5	39	−5	−38
*T*_d_ (°C)	312	281	280	274

*M*_n_ values were obtained by GPC using polystyrene calibration. Silyl-protected polymers were measured under standard THF GPC conditions, whereas the deprotected hydroxylated polymers were measured by GPC using DMF as the eluent. For the deprotected polymers, the percentage shown in parentheses after *M*_n_ (e.g., 7500 (−61.7%)) denotes the relative decrease in *M*_n_ with respect to its silyl-protected precursor: Δ *M*_n_ (%) = (*M*_n_, protected − *M*_n_, deprotected)/*M*_n_, protected × 100%. *T*_g_ is the glass transition temperature determined by DSC (second heating scan). *T*_d_ is the temperature at 5 wt% mass loss in TGA under N_2_.

**Table 2:** Solubility Characteristics of Poly(hydroxyalkyl propenyl ether)s and related polymers.

	Poly(HPPE)	Poly(HBPE)	Poly(TBDPSBPE)	Poly(HBVE) [10]
Solvent	Polymer Solubility			
H_2_O (5 °C)	○	×	×	○
H_2_O (25 °C)	×	×	×	○
Methanol, DMSO	○	○	×	○
Acetone	×	×	×	×
CHCl_3_, THF, toluene, diethyl ether	×	×	○	×

The solubility of the polymers was measured with 0.25 wt% solutions at room temperature. ○ = soluble; × = insoluble.

## Data Availability

The original contributions presented in this study are included in the article/Supplementary Material. Further inquiries can be directed to the corresponding author.

## Supplementary Materials

The following supporting information can be downloaded at: https://www.mdpi.com/article/10.3390/molecules30224345/s1, the synthesis and characterization of BMSBPE, TIPSBPE, TBDPSPPE, and TBDPSBVE. Figure S1: ^1^H NMR spectrum of 4-hydroxybutyl allyl ether (HBAE) in CDCl_3_; Figure S2: (a) ^1^H NMR spectrum and (b) ^13^C NMR spectrum of HBPE in CDCl_3_; Figure S3: (a) ^1^H NMR spectrum and (b) ^13^C NMR spectrum of *tert*-butyldiphenylsiloxybutyl vinyl ether (TBDPSBPE) in CDCl_3_; Figure S4: (a) ^1^H NMR spectrum and (b) ^13^C NMR spectrum of BMSBPE in CDCl_3_; Figure S5: (a) ^1^H NMR spectrum and (b) ^13^C NMR spectrum of TIPSBPE in CDCl_3_; Figure S6: ^1^H NMR spectrum of HPAE in CDCl_3_; Figure S7: ^1^H NMR spectrum of HPPE in CDCl_3_; Figure S8: (a) ^1^H NMR spectrum and (b) ^13^C NMR spectrum of TBPSPPE in CDCl_3_; Figure S9: (a) ^1^H NMR spectrum and (b) ^13^C NMR spectrum of TBPSBVE in CDCl_3_; Figure S10: Monomer-addition experiment for the polymerization of TIPSBPE with IBEA/Et_1.5_AlCl_1.5_/SnCl_4_ in toluene at −80 °C ([monomer]_0_ = 0.6 M, [monomer]_add_ = 0.6 M, [IBEA]_0_ = 4.0 mM, [Et_1.5_AlCl_1.5_]_0_ = 4.0 mM, [SnCl_4_]_0_ = 5.0 mM, [AcOEt]_0_ = 1.0 M); Figure S11: GPC traces of (a) poly(TBDPSPPE) prepared using IBEA/Et_1.5_AlCl_1.5_/SnCl_4_ in toluene at −80 °C in the presence of AcOEt ([monomer]_0_ = 0.6 M, [IBEA]_0_ = 4.0 mM, [Et_1.5_AlCl_1.5_]_0_ = 4.0 mM, [SnCl_4_]_0_ = 5.0 mM, [AcOEt]_0_ = 1.0 M); Figure S12: ^1^H NMR spectrum of poly(HPPE) in DMSO-*d*_6_; Figure S13: GPC traces (DMF eluent, polystyrene calibration) of (a) poly(HBVE), (b) poly(HBPE), and (c) poly(HPPE) after desilylation; Figure S14: Glass transition temperature of poly(β-methyl vinyl ether)s and poly(vinyl ether)s with a bulky silyl protecting group or a hydroxy group; Figure S15: 5% weight loss temperature of various poly(β-methyl vinyl ether)s and poly(vinyl ether)s with a bulky silyl protecting group or a hydroxy group.

## Abbreviations

The following abbreviations are used in this manuscript:

TBDPSBPE	Tert-butyldiphenylsiloxybutyl propenyl ether
NBPE	n-butyl propenyl ether
IBPE	Isobutyl propenyl ether
EPE	Ethyl propenyl ether
TBPE	Tert-butyl propenyl ether
HBPE	4-hydroxybutyl propenyl ether
BMSBPE	Tert-butyldimethylsiloxybutyl propenyl ether
TIPSBPE	Triisopropylsiloxybutyl propenyl ether
LCST	Lower critical solution temperature

## Appendix A

The polymerization of 1-adamantane propenyl ether (1-AdPE) was examined at −80 °C in toluene using the IBEA/Et_1.5_AlCl_1.5_/SnCl_4_/AcOEt initiator system; however, the resulting polymer was only partially soluble in the organic solvent. [1-AdPE]_0_ = 0.6 M, [IBEA]_0_ = 4.0 mM, [Et_1.5_AlCl_1.5_]_0_ = 4.0 mM, [SnCl_4_]_0_ = 50 mM, [AcOEt]_0_ = 1.0 M.
