# Supplementary material for: Living Cationic Polymerization of Silyl-Protected β-Methyl Vinyl Ethers (Propenyl Ethers): Synthesis of Hydroxy-Functional Polymers with High Tg and LCST-Type Thermoresponse
Source: Molecules. 2025 Nov 10;30(22):4345. doi: 10.3390/molecules30224345 (PMC12654800; doi:10.3390/molecules30224345)
Supplement: Supplementary file 1 [file molecules-30-04345-s001.zip › molecules-3952255-supplementary.docx]

Supporting Information

Article

Living Cationic Polymerization of Silyl-Protected β-Methyl Vinyl Ethers (Propenyl Ethers): Synthesis of Hydroxy-Functional Polymers with High *T*_g_ and LCST-Type Thermoresponse

Kohei Watanabe, Ryuya Yamada and Takeshi Namikoshi ^*^

School of Earth, Energy and Environmental Engineering, Faculty of Engineering, Kitami Institute of Technology, Kitami 090-8507, Japan

***** Correspondence: takenami@mail.kitami-it.ac.jp; Tel.: +81-157-26-9433

*Synthesis of tert-butyldimethylsiloxybutyl propenyl ether (BMSBPE)*

BMSBPE was synthesized by reacting 4-hydroxybutyl propenyl ether (HBPE) with *tert*-butyldimethylchlorosilane [1]. A solution of *tert*-butyldimethylchlorosilane (9.19 g, 0.061 mol) in *N*,*N*-dimethylformamide (DMF; 13.8 mL) was added dropwise to a mixture of HBPE (7.94 g, 0.061 mol), imidazole (9 g, 0.132 mol), and DMF (13.8 mL) at 0 °C under nitrogen. The mixture was stirred at room temperature for 6 h and then washed with water. The product was isolated by column chromatography using a mixture of ethyl acetate and hexane (1/30, v/v) and then distilled over calcium hydride under reduced pressure to obtain BMSBPE as a colorless liquid (yield: 12.86 g, 72%; density: 0.8372 g/mL; bp: 64 °C/3 mmHg). ^1^H NMR (CDCl_3_, ppm): 0.04 (s, 6H, –Si–(CH_3_)_2_), 0.89 (s, 9H, –Si–C(CH_3_)_3_), 1.54, 1.57 (m, 3H, CH_3_CH=CH–), 1.61 (m, 2H, –O–CH_2_CH_2_CH_2_CH_2_–O–Si–), 1.67 (m, 2H, –O–CH_2_CH_2_CH_2_CH_2_–O–Si–), 3.64, 3.73 (m, 4H, –O–CH_2_CH_2_CH_2_CH_2_–O–Si–), 4.36, 4.75 (m, 1H, CH_3_CH=CH–O–), 5.93, 6.20 (dd, 1H, CH_3_CH=CH–O–) (Figure S4a); ^13^C NMR (CDCl_3_, δ): -5.20 (–Si–(CH_3_)_2_), 9.33, 12.7 (CH_3_CH=CH–), 25.8–29.4 (–O–CH_2_CH_2_CH_2_CH_2_–O–Si–), 26.1 (–Si–C(CH_3_)_3_), 63.0 (–O–CH_2_CH_2_CH_2_CH_2_–O–Si–), 69.1, 72.0 (–O–CH_2_CH_2_CH_2_CH_2_–O–Si–), 98.4, 100.9 (CH_3_CH=CH–O–), 145.7, 146.8 (CH_3_CH=CH–O–) (Figure S4b); HRMS (EI): *m*/*z* calculated for C_9_H_19_O_2_Si [M-CH(CH_3_)_2_]^+^: 187.11543, found 187.11551.

*Synthesis of triisopropylsiloxybutyl propenyl ether (TIPSBPE)*

TIPSBPE was synthesized by reacting HBPE with triisopropylchlorosilane [2]. A solution of triisopropylchlorosilane (7.63 mL, 0.036 mol) in DMF (11.2 mL) was added dropwise to a mixture of HBPE (4.70 g, 0.036 mol), imidazole (4.42 g, 0.065 mol), and DMF (11.2 mL) at 0 °C under nitrogen. The mixture was stirred at room temperature for 6 h and then washed with water. The product was isolated by column chromatography using a mixture of ethyl acetate and hexane (1/30, v/v) to give TIPSBPE as a colorless liquid (isolated yield: 65%; density: 0.9011 g/mL). ^1^H NMR (CDCl_3_, ppm): 1.06 (s, 21H, –Si–(CH(CH_3_)_2_)_3_), 1.54, 1.58 (m, 3H, CH_3_CH=CH–), 1.62 (m, 2H, –O–CH_2_CH_2_CH_2_CH_2_–O–Si–), 1.71 (m, 2H, –O–CH_2_CH_2_CH_2_CH_2_–O–Si–), 3.65, 3.72 (m, 4H, –O–CH_2_CH_2_CH_2_CH_2_–O–Si–), 4.36, 4.75 (m, 1H, CH_3_CH=CH–O–), 5.93, 6.21 (dd, 1H, CH_3_CH=CH–O–) (Figure S5a); ^13^C NMR (CDCl_3_, ppm): 9.30, 12.7 (CH_3_CH=CH–), 12.1 (–Si–(CHCH_3_CH_3_)_3_), 18.1 (–Si–(CH(CH_3_)_2_)_3_), 26.1, 26.5 (–O–CH_2_CH_2_CH_2_CH_2_–O–Si–), 26.6 (–O–CH_2_CH_2_CH_2_CH_2_–O–Si–), 63.2 (–O–CH_2_CH_2_CH_2_CH_2_–O–Si–), 69.1, 72.0 (–O–CH_2_CH_2_CH_2_CH_2_–O–Si–), 98.4, 100.9 (CH_3_CH=CH–O–), 145.7, 146.8 (CH_3_CH=CH–O–) (Figure S5b); HRMS (EI): *m*/*z* calculated for C_13_H_27_O_2_Si [M-(CHCH_3_CH_3_)_3_]^+^: 243.17803, found 243.17776.

*Synthesis of 4-hydroxy propyl allyl ether (HPAE)*

HPAE was synthesized by the reaction of 1,3-propanediol with allyl bromide [3]. 1,3-Propanediol l (7.97 mL, 0.11 mol), allyl bromide (9.59 mL, 0.11 mol), TBAB (0.70 g, 2.20 mmol), sodium hydroxide (4.40 g, 0.11 mol), and toluene (30 mL) were added to a 200 mL three-necked round-bottom flask fitted with a reflux condenser, which was then purged with nitrogen. After heating at 70 °C for 10 h with stirring, the reaction mixture was washed with water. The aqueous phase was then extracted with toluene, and the combined organic layers were dried and concentrated to afford HPAE as a colorless transparent liquid (yield: 9.67 g, 76%). ^1^H NMR (CDCl_3_, ppm): 1.83, 1.87 (m, 2H, –O–CH_2_CH_2_CH_2_–OH), 2.75 (s, 1H, –O–CH_2_CH_2_CH_2_CH_2_–OH), 3.52, 3.60, 3.75 (t, 4H, –O–CH_2_CH_2_CH_2_–OH), 3.97 (t, 2H, CH_2_=CHCH_2_–O–), 5.17, 5.26 (m, 2H, CH_2_=CHCH_2_–O–), 5.90 (m, 1H, CH_2_=CHCH_2_–O–) (Figure S6).

*Synthesis of hydroxypropyl propenyl ether (HPPE)*

HPPE was synthesized by a ruthenium-catalyzed reaction of HPAE with methanol [3]. An autoclave reactor with polytetrafluoroethylene-lined vessel was charged with HPAE (9.67 g, 0.083 mol), methanol (10.1 mL, 0.249 mol), sodium carbonate (0.44 g, 4.15 mmol), and RuCl_2_(PPh_3_)_3_ (0.80 g, 0.83 mmol) and then purged with nitrogen. The mixture was heated at 120 °C for 3 h with stirring. After filtering the reaction mixture to remove sodium carbonate and RuCl_2_(PPh_3_)_3_, the product was isolated by column chromatography using a mixture of AcOEt and hexane (1/1, v/v) and then distilled over calcium hydride under reduced pressure to obtain HPPE as a colorless liquid (yield: 5.00 g, 52%; bp: 28 °C/1 mmHg). ^1^H NMR (CDCl_3_, ppm): 1.55-1.58 (d, 3H, CH_3_CH=CH–O–), 1.89 (m, 2H, –O–CH_2_CH_2_CH_2_–OH), 3.76-3.81, 3.89 (m, 4H, –O–CH_2_CH_2_CH_2_–OH), 4.42, 4.80 (m, 1H, CH_3_CH=CH–O–), 5.95, 6.22 (dd, 1H, CH_3_CH=CH–O–) (Figure S7). Notably, after isomerization and purification, the allyl-ether signals (CH_2_=CH–CH₂–O–; ^1^H NMR) were no longer observed, consistent with isomerization to the propenyl ether structure prior to silylation and polymerization. The product contained a mixture of Z/E isomers (~58:42 based on the vinyl proton NMR signals). No attempt was made to separate these isomers, and the mixture was used as-is in subsequent reactions.

*Synthesis of tert-butyldiphenylsiloxypropyl propenyl ether (TBDPSPPE)*

TBDPSPPE was synthesized by reacting HPPE with *tert*-butyldiphenylchlorosilane [2]. A solution of *tert*-butyldiphenylchlorosilane (13.6 mL, 0.053 mol) in DMF (10 mL) was added dropwise to a mixture of HPPE (6.14 g, 0.053 mol), imidazole (8.5 g, 0.125 mol), and DMF (12 mL) at 0 °C under nitrogen. The mixture was stirred at room temperature for 6 h and then washed with water. The crude monomer was isolated by column chromatography using a mixture of AcOEt and hexane (1/20, v/v) and then distilled over calcium hydride under reduced pressure to obtain TBDPSPPE as a colorless liquid (isolated yield: 53%; density: 0.9684 g/mL; bp: 140 °C/2 mm Hg). The purified monomer retained the original Z/E isomer ratio (~60:40) of HPPE, as evidenced by consistent ¹H NMR signals. ^1^H NMR (CDCl_3_, ppm): 1.09 (s, 9H, –Si–C(CH_3_)_3_), 1.57, 1.58 (t, 3H, CH_3_CH=CH–O–), 1.89 (m, 2H, –O–CH_2_CH_2_CH_2_–O–Si–), 3.79, 3.92 (m, 4H, –O–CH_2_CH_2_CH_2_–O–Si–), 4.39, 4.78 (t, 1H, CH_3_CH=CH–O–), 5.98, 6.23 (dd, 1H, CH_3_CH=CH–O–), 7.38-7.45, 7.68 (m, 10H, –Si–(C_6_H_5_)_2_) (Figure S8a); ^13^C NMR (CDCl_3_, ppm): 9.35, 12.77 (CH_3_CH=CH–O–), 19.3, 26.97 (–Si–C(CH_3_)_3_), 60.32 (–O–CH_2_CH_2_CH_2_–O–Si–), 65.89, 68.73 (–O–CH_2_CH_2_CH_2_–O–Si–), 98.55, 100.9 (CH_3_CH=CH–O–), 127.8-135.7 (–Si– (C_6_H_5_)_2_), 145.8, 146.8 (CH_3_CH=CH–O–) (Figure S8b).

*Synthesis of tert-butyldiphenylsiloxybutyl vinyl ether (TBDPSBVE)*

TBDPSBVE was synthesized by reacting 4-hydroxybutyl vinyl ether (HBVE) with *tert*-butyldiphenylchlorosilane [2]. A solution of *tert*-butyldiphenylchlorosilane (8.72 mL, 0.034 mol) in DMF (10 mL) was added dropwise to a mixture of HBVE (4.88 g, 0.042 mol), imidazole (5 g, 0.074 mol), and DMF (10 mL) at 0 °C under nitrogen. The mixture was stirred at room temperature for 6 h and then washed with water. The crude monomer was distilled over calcium hydride under reduced pressure to give TBDPSBVE as a colorless liquid (isolated yield: 81%; density: 0.9971 g/mL; bp: 149 °C/2 mm Hg). ^1^H NMR (CDCl_3_, ppm): 1.07 (s, 9H, –Si–C(CH_3_)_3_), 1.67 (m, 2H, –O–CH_2_CH_2_CH_2_CH_2_–O–Si–), 1.78 (m, 2H, –O–CH_2_CH_2_CH_2_CH_2_–O–Si–), 3.68-3.72 (m, 4H, –O–CH_2_CH_2_CH_2_CH_2_–O–Si–), 3.99, 4.17 (dd, 2H, CH_2_=CH–O–), 6.47 (q, 1H, CH_2_=CH–O–), 7.38-7.45, 7.66, 7.99 (m, 10H, –Si–(C_6_H_5_)_2_) (Figure S9a); ^13^C NMR (CDCl_3_, ppm): 19.1-30.9 (–O–CH_2_CH_2_CH_2_CH_2_–O–Si–), 26.8 (–Si–C(CH_3_)_3_), 63.4 (–O–CH_2_CH_2_CH_2_CH_2_–O–Si–), 67.8 (–O–CH_2_CH_2_CH_2_CH_2_–O–Si–), 86.2 (CH_2_=CH–O–), 127.6-135.5 (–Si–(C_6_H_5_)_2_), 151.8 (CH_2_=CH–O–) (Figure S9b); HRMS (EI): m/z calculated for C_15_H_21_O_2_Si [M-(CCH_3_)_3_]^+^: 297.13108, observed 297.13080.

*Synthesis of poly(TBDPSPPE)*

Polymerization of TBDPSPPE was carried out at -30 °C in toluene in the presence of ethyl acetate (AcOEt) ([TBDPSBPE]_0_ = 0.6 M, [IBEA]_0_ = 4.0 mM, [Et_1.5_AlCl_1.5_]_0_ = 4.0 mM, [SnCl_4_]_0_ = 5.0 mM, and [AcOEt]_0_ = 1.0 M). Figure S11 shows the GPC chromatogram of the obtained poly(TBDPSPPE).

*Synthesis of poly(HPPE)*

The synthesis of poly(HPPE) was carried out by desilylation of a precursor polymer, similar to the synthesis of poly(HBPE). The ^1^H NMR spectrum of the resulting polymer is shown in Figure S12.

**Figure S1.** ^1^H NMR spectrum of 4-hydroxybutyl allyl ether (**HBAE**) in CDCl_3_.

**Figure S2.** (a) ^1^H NMR spectrum and (b) ^13^C NMR spectrum of **HBPE** in CDCl_3_.

**Figure S3.** (a) ^1^H NMR spectrum and (b) ^13^C NMR spectrum of **TBDPSBPE** in CDCl_3_.

**Figure S4.** (a) ^1^H NMR spectrum and (b) ^13^C NMR spectrum of **BMSBPE** in CDCl_3_.

**Figure S5.** (a) ^1^H NMR spectrum and (b) ^13^C NMR spectrum of **TIPSBPE** in CDCl_3_.


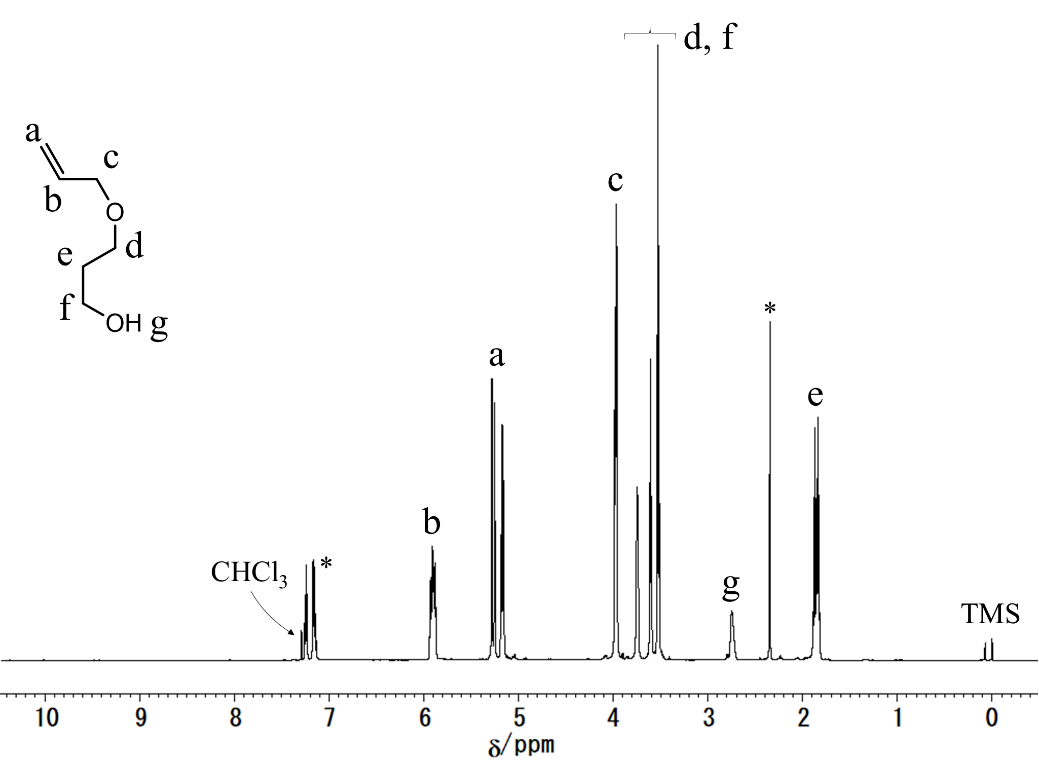


toluene

toluene

**Figure S6.**  ^1^H NMR spectrum of **HPAE** in CDCl_3_.


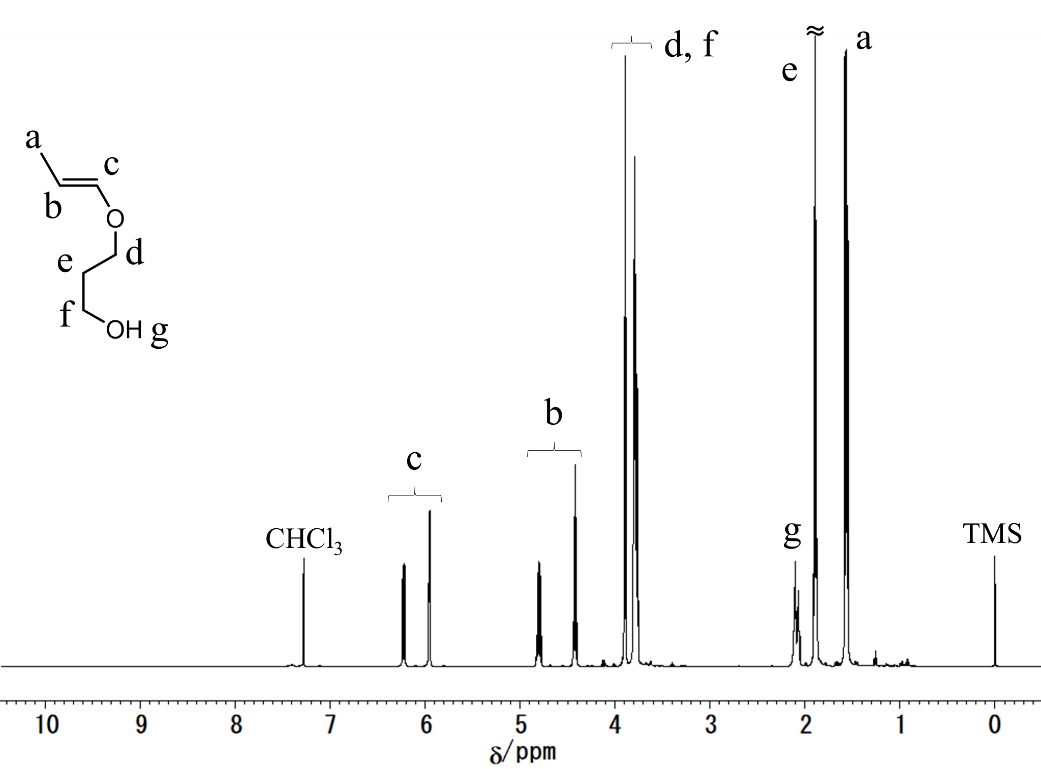


**Figure S7.**  ^1^H NMR spectrum of **HPPE** in CDCl_3_.


**Figure S8.** (a) ^1^H NMR spectrum and (b) ^13^C NMR spectrum of **TBDPSPPE** in CDCl_3_.

**Figure S9.** (a) ^1^H NMR spectrum and (b) ^13^C NMR spectrum of **TBDPSBVE** in CDCl_3_.


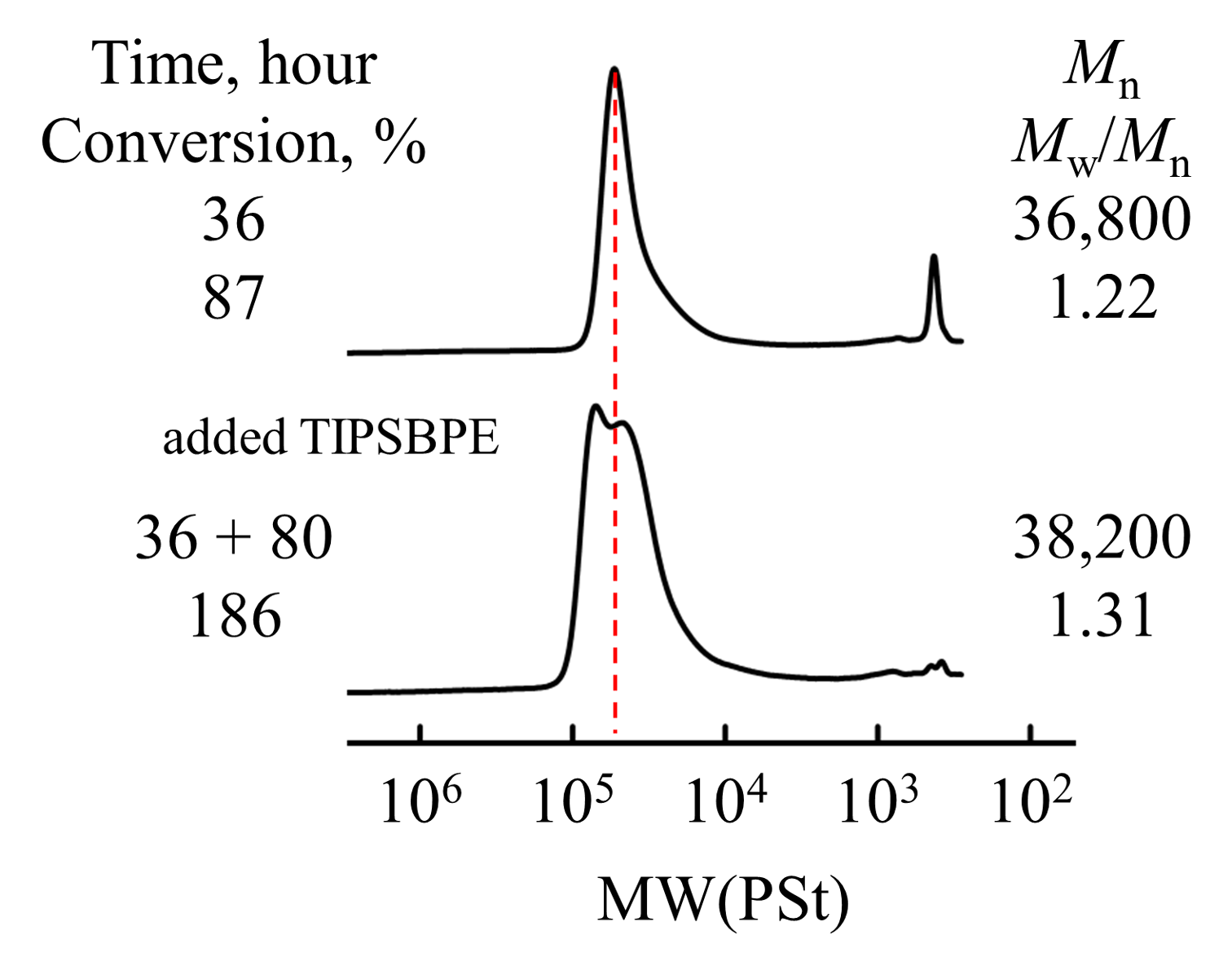


**Figure S10.** Monomer-addition experiment for the polymerization of **TIPSBPE** with IBEA/Et_1.5_AlCl_1.5_/SnCl_4_ in toluene at -80 °C ([monomer]_0_ = 0.6 M, [monomer]_add_ = 0.6 M, [IBEA]_0_ = 4.0 mM, [Et_1.5_AlCl_1.5_]_0_ = 4.0 mM, [SnCl_4_]_0_ = 5.0 mM, [AcOEt]_0_ = 1.0 M).

**Figure S11.** GPC traces of (a) poly(**TBDPSPPE**) prepared using IBEA/Et_1.5_AlCl_1.5_/SnCl_4_ in toluene at -80 °C in the presence of AcOEt ([monomer]_0_ = 0.6 M, [IBEA]_0_ = 4.0 mM, [Et_1.5_AlCl_1.5_]_0_ = 4.0 mM, [SnCl_4_]_0_ = 5.0 mM, [AcOEt]_0_ = 1.0 M).

**Figure S12.** ^1^H NMR spectrum of poly(**HPPE**) in DMSO-*d*_6_.

**Figure S13.** GPC traces (DMF eluent, polystyrene calibration) of (a) poly(**HBVE**), (b) poly(**HBPE**), and (c) poly(**HPPE**) after desilylation. Each sample elutes as a single, unimodal peak.


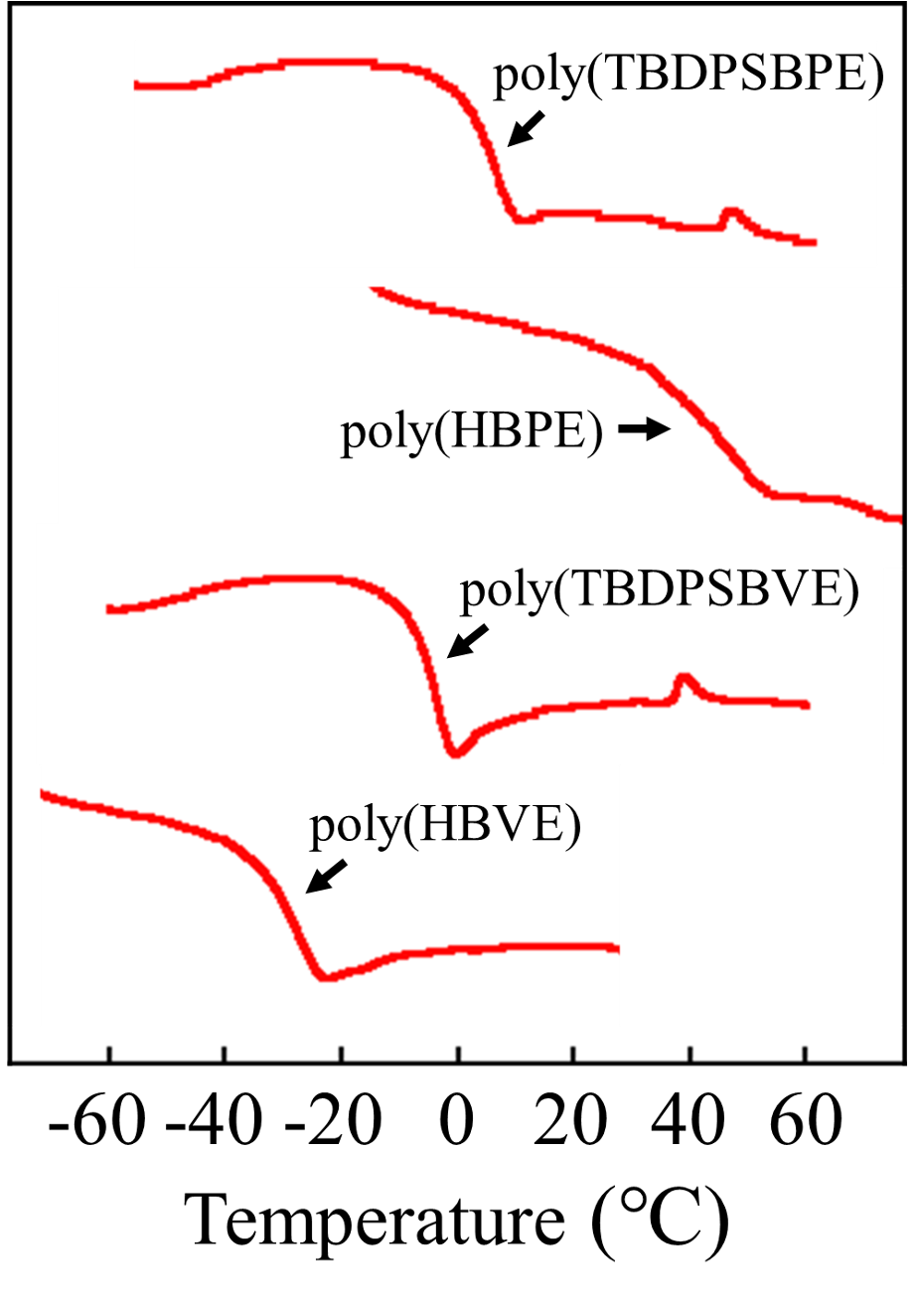


**Figure S14.** Glass transition temperatures of poly(propenyl ether)s and related poly(vinyl ether)s, comparing polymers with bulky silyl-protected side chains and the corresponding deprotected polymers bearing pendant hydroxy groups.

**Figure S15.** 5% weight-loss temperatures of poly(propenyl ether)s and related poly(vinyl ether)s, comparing polymers with bulky silyl-protected side chains and their deprotected analogues bearing pendant hydroxy groups.

References and notes

1. Sugihara, S.; Hashimoto, K.; Matsumoto, Y.; Kanaoka, S.; Aoshima, S., Thermosensitive Polyalcohols: Synthesis via Living Cationic Polymerization of Vinyl Ethers with a Silyloxy Group. *Journal of Polymer Science, Part A: Polymer Chemistry* **2003,** 41, (21), 3300–3312.

2. Corey, E. J.; Venkateswarlu, A., Protection of hydroxyl groups as tert-butyldimethylsilyl derivatives. *Journal of the American Chemical Society* **1972,** 94, (17), 6190–6191.

3. Krompiec, S.; Penczek, R.; Penkala, M.; Krompiec, M.; Rzepa, J.; Matlengiewicz, M.; Jaworska, J.; Baj, S., A selective convenient ruthenium-mediated synthesis of mixed acetals. *Journal of Molecular Catalysis A: Chemical* **2008,** 290, (1-2), 15–22.
